# Feasibility and Acceptability of a Counseling- and mHealth-Based Physical Activity Intervention for Pregnant Women With Diabetes: The Fit for Two Pilot Study

**DOI:** 10.2196/18915

**Published:** 2020-10-21

**Authors:** Britta Larsen, Stephanie Micucci, Sheri J Hartman, Gladys Ramos

**Affiliations:** 1 Department of Family Medicine & Public Health University of California, San Diego San Diego, CA United States; 2 Department of Obstetrics, Gynecology, and Reproductive Sciences University of California, San Diego San Diego, CA United States

**Keywords:** exercise, behavioral medicine, mHealth, gestational diabetes, type 2 diabetes, mobile phone

## Abstract

**Background:**

Diabetes during pregnancy poses serious health risks to both mother and child. Regular physical activity can reduce these risks, yet few clinic-based interventions of physical activity for pregnant women with diabetes have been attempted.

**Objective:**

The purpose of this single-arm pilot trial is to assess the feasibility and acceptability, and explore the potential efficacy of a counseling- and mobile health–based physical activity intervention for pregnant women with diabetes.

**Methods:**

Participants (N=17) who had type 2 or gestational diabetes, could read and speak in English or Spanish, and were between 10 and 27 weeks of gestation were recruited from the University of California San Diego Diabetes and Pregnancy Program. Participants engaged in a one-on-one counseling and goal-setting session immediately following a clinic visit with their physician. They were given a Fitbit and shown how to use the Fitbit app, including entering personalized step goals, and were encouraged to build up to 10,000 daily steps. Daily steps were recorded for 12 weeks, until they were 36 weeks’ gestation, or until 1 week before they gave birth, whichever came first. Feasibility was measured by recruitment, retention, and adherence, and acceptability was measured using consumer satisfaction questionnaires and follow-up interviews. Potential efficacy was explored by examining changes in daily steps over time.

**Results:**

The participants were primarily Hispanic (13/17, 76%), had public insurance (15/17, 88%), and had type 2 diabetes (12/17, 71%). Of the 17 patients who began the intervention, 76% (13/17) completed a follow-up visit, and 71% (12/17) continued wearing the Fitbit regularly after 8 weeks in the intervention. Adherence in wearing the Fitbit was relatively high, with a median wear adherence of 90% of days. The intervention was generally well accepted, with 85% (11/13) indicating that they were motivated to exercise more following the counseling session, 85% (11/13) indicating that the Fitbit helped increase their activity, and 92% (12/13) recommending the program overall. Mean daily steps increased from baseline (mean 6122, SD 2439) to week 3 (mean 6269, SD 2166) and then decreased through week 12 (mean 4191, SD 2228).

**Conclusions:**

High acceptability, retention, and adherence suggest that this may be a promising approach to delivering a simple, low-burden intervention in a clinical setting to a high-risk, underserved population. A randomized controlled trial is needed to determine whether this approach is effective in slowing the reduction in activity typically seen throughout pregnancy.

**Trial Registration:**

ClinicalTrials.gov NCT03302377; https://clinicaltrials.gov/ct2/show/NCT03302377

## Introduction

Diabetes during pregnancy can have serious health consequences for both mother and child. Women who experience type 2 diabetes (T2D) during pregnancy are less likely to return to prepregnancy weight and are more likely to experience glycemic dysregulation following pregnancy [1]. Infants exposed to T2D in utero have approximately 9 times higher risk of perinatal mortality and are at a higher risk for congenital malformation and developing obesity and T2D later in life [2-4]. Given the rapidly rising rates of diabetes among young adults of childbearing age [5] and estimates that diabetes prevalence will more than double in the United States by 2050 [6] and more than quadruple in youth [7], this constitutes a critical public health issue.

Physical activity has robustly been shown to prevent T2D [8], and limited clinical trial research suggests that it can normalize glucose and prevent insulin use in pregnant women with diabetes [9-11]. The American College of Obstetrics and Gynecologists concordantly now recommends an individualized exercise prescription for all pregnant women with diabetes [12]. Intervening on physical activity during pregnancy is much needed, as activity typically declines during pregnancy [13,14], particularly for women who were initially underactive [15]. Delivery of prenatal exercise counseling, however, is inconsistent and often absent, as most providers lack time or training to provide all the tools necessary to help patients change physical activity [16,17].

Advances in technology, however, have made the dissemination of individualized physical activity interventions more feasible [18,19]. Commercial wearable activity trackers and accompanying smartphone apps use features that have consistently been associated with successful behavioral changes, such as goal setting, self-monitoring, reminders, and social accountability [20]. Accordingly, interventions combining wearable trackers with brief counseling have shown significant improvements in physical activity [21,22]. As wearable devices are not only effective but also low cost relative to other clinical interventions, they could be especially appropriate for incorporation into clinical care, although studies of the efficacy, feasibility, and acceptability of using wearable devices with clinical populations remain sparse. Some small pilot studies have found that using consumer wearable devices may be feasible and effective in increasing physical activity in pregnant women [23]; however, no such studies have been performed to date in pregnant women with diabetes.

Given the growing rates of diabetes in pregnancy and the significant risks associated with this condition, this is a pressing public health issue, and developing effective, low-cost physical activity interventions with potential for implementation in clinical and community settings is essential for promoting healthy pregnancies and infants. Therefore, we designed a low-cost, low-burden physical activity intervention combining an in-person counseling session with the use of a consumer wearable tracker and mobile phone app. The purpose of this study is to evaluate the feasibility and acceptability of this physical activity intervention for pregnant women with diabetes.

## Methods

### Study Overview

The *Fit for Two* study was a single-arm pilot trial to assess the feasibility and acceptability of a physical activity intervention in a clinical setting for pregnant women with T2D or gestational diabetes (GD). The intervention comprised an in-person counseling session followed by the use of a Fitbit wrist monitor and app for 8-12 weeks to reinforce key behavior change strategies. Physical activity was measured throughout the trial via Fitbit monitors, and feedback on the intervention was provided at the study conclusion via consumer satisfaction questionnaires and individual interviews.

### Participants

Participants were pregnant women receiving prenatal care at the University of California San Diego (UCSD) Diabetes and Pregnancy Program (DAPP). Women were eligible to enroll if they (1) were currently pregnant and between 10 and 27 weeks of gestation, (2) were aged 18 years or older, (3) had daily access to a smartphone, (4) were underactive (engaged in less than 100 min per week of at least moderate-intensity activity), (5) could read and speak in English or Spanish, and (6) were diagnosed with either T2D or GD. Patients with T2D were those who had previously been diagnosed with T2D before pregnancy, whereas those with GD were diagnosed with diabetes during pregnancy. Women were ineligible if they had any condition that would make exercise unsafe as determined by their physician or if they were concurrently participating in another behavioral or pharmaceutical trial. The study was approved by the UCSD institutional review board, and all participants provided written informed consent.

### Recruitment

Recruitment occurred during DAPP clinic visits. Using electronic medical records, Health Insurance Portability and Accountability Act–certified study staff checked weekly patient visit schedules to identify patients who met the eligibility criteria. The patient’s physician was asked to confirm whether she could safely participate in a physical activity study or whether she had any contraindications. At the end of their clinic visits, potentially eligible patients were asked by the attending physician whether they were interested in learning about a study that could help them increase their physical activity. Study staff was on-site to discuss the study with interested participants and complete a screening interview to confirm eligibility.

### Protocol Overview

**Figure 1:**
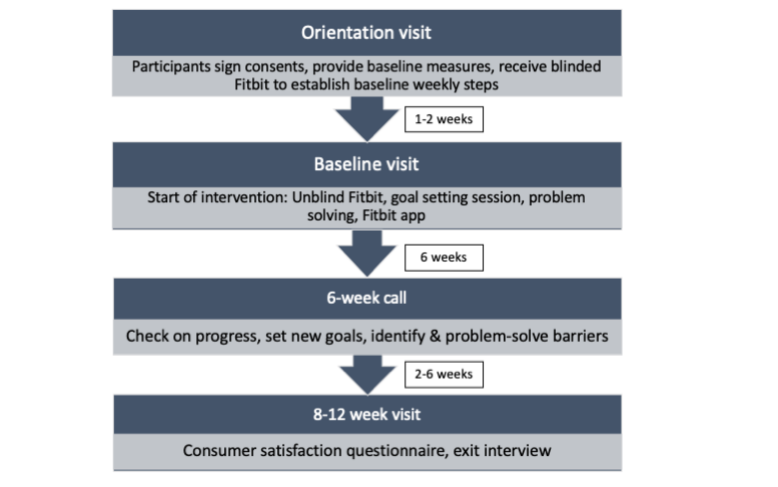
Intervention timeline.

As most patients in the DAPP clinic have prenatal care appointments scheduled every 1-3 weeks for glucose review, study visits were designed to coincide with the scheduled clinic visits. Participants attended an orientation visit in an exam room at the clinic to learn more details about the study and give informed consent. Participants who were Spanish-dominant met with a bilingual researcher and completed consent and all forms and study visits in Spanish. They were then given a blinded Fitbit Alta HR wrist-worn activity monitor to wear for 1 week to establish baseline steps and activity (see the *Measures* section). Following their next prenatal care visit 1-3 weeks later, the participants started the physical activity intervention. Figure 1 shows a timeline of the intervention activities.

### Intervention Description

The *Fit for Two* intervention was centered on evidence-based behavior change strategies such as goal setting, self-monitoring, accountability, and social support. The intervention comprised an in-person counseling session to teach key behavior change constructs and initiate behavior change, followed by continued support through the Fitbit app and activity monitor to reinforce behavior change strategies. This allowed for both depth and breadth of the intervention content with relatively little staff time or clinic space required.

During the in-person counseling session at the start of the intervention, participants learned more about the importance of physical activity during pregnancy and the types and amounts of activity they should engage in. The interventionist then unblinded the participant’s Fitbit data from the previous week to review their current steps and activity. Using the principles of motivational interviewing, the interventionist helped the participant set her personal goals for daily steps, identify potential barriers to achieving goals, and outline problem-solving strategies. The data were used to set realistic goals for daily steps and discuss potential barriers and problem solving around them using principles of motivational interviewing. The study staff then helped participants learn to use the Fitbit monitor and app to support self-monitoring and get feedback toward goals and reinforcement when goals are met through congratulatory messages, visual displays on the app, and a digital firework display on the wrist monitor. Participants were encouraged to set their own new goals regularly to gradually build up to 10,000 steps at whatever pace was right for them and keep their physician informed of their progress by reporting their average daily steps per week in their weekly email reporting daily glucose levels. For the duration of the intervention (8-12 weeks depending on the timing of pregnancy), participants were instructed to wear the Fitbit wrist monitor daily and synchronize it with the Fitbit app at least weekly. Fitabase (Small Steps Lab, LLC), a third-party software platform that extracts the Fitbit data from authorized users in real time, was used by the study staff to monitor syncing and provided text reminders if participants had not synced their Fitbit monitor with the app for more than 2 weeks.

Participants also received a brief (10-min) phone call after 6 weeks to review progress and report any problems.

### Measures

Basic demographics were collected via questionnaires at baseline and included age, race, ethnicity, education, employment, income, marital status, and the number of children. Medical data were collected from participants’ electronic medical records both at baseline and follow up and included current weight, weight gain since pregnancy, week of gestation, resting blood pressure (systolic and diastolic), hemoglobin A_1c_ (HbA_1c_), and nonfasting glucose.

#### Consumer Satisfaction

We used a questionnaire to assess the feasibility and acceptability of various intervention components, including the goal-setting session, educational material, the Fitbit monitor, and the smartphone app. It was available in English or Spanish; the measure has been translated and back-translated in Spanish and adapted from previous interventions with Spanish-speaking individuals [24]. Participants were also invited to engage in a one-on-one follow-up interview to give more detailed feedback about their experience in the program.

#### Physical Activity

Daily steps were measured throughout the study using the Fitbit Alta HR monitor. It is a wrist-worn, combined sensing (ie, accelerometry and photoplethysmography) activity tracker that measures physical activity (ie, intensity, energy expenditure, bouts of exercise, steps, and distance traveled) at varying resolutions ranging from 1 second to 1 min daily. The Fitbit app automatically syncs data from the wrist monitor to a smartphone app via Bluetooth, which was collected and stored through Fitabase. A valid day of Fitbit wear was defined as ≥600 mins (10 hours) of heart rate data, or for participants with inconsistent or missing heart rate data, ≥6000 steps. A partial wear day was defined as any day with ≥1000 steps. The literature has shown that days with <1000 steps are unlikely to show actual wear [25]. To prevent feedback from influencing baseline activity, the displays on the wrist monitor and app were then blinded by removing all information tiles from the app and setting the wrist monitor to only show time, battery life, and distance. The Fitbit has shown excellent reliability and validity for measuring daily steps [26].

Participants were also given an ActiGraph GT3X+ hip-worn accelerometer to wear for 1 week at baseline and follow up to provide a measure of activity at various intensities. However, a high percentage of participants (13/17, 76%) commented that the belt-worn accelerometer was markedly uncomfortable during pregnancy and requested to stop wearing it; thus, this measure was discontinued.

### Analysis

Feasibility was defined by 3 factors: (1) recruitment if at least 50% of the women who expressed an interest in the study enrolled, (2) retention if at least 75% of the participants who attended a baseline session completed the follow-up assessment and/or interview, and (3) adherence if the participants wore and synced the Fitbit on at least 75% of days. Acceptability was defined as at least 75% of the respondents indicated that they were *satisfied* or *very satisfied* with the intervention overall and individual intervention components.

As the primary aim was assessing feasibility and acceptability, we did not calculate the power to determine efficacy, but we did evaluate changes in activity from baseline to follow up (week 12) to explore the potential implications for efficacy. Daily steps were converted into average daily steps for the baseline period (the week immediately following the orientation visit) and each week of the trial following the baseline intervention visit. We used 2 separate average daily step variables to plot activity and explore trajectories of behavior change across weeks in the intervention and across gestational weeks: (1) days with any partial Fitbit wear time and (2) days where the Fitbit was worn the entire day. For each of these figures, we present group means plotted with a local polynomial regression fitting or *loess* function to examine nonlinear changes across follow up. Analyses were performed using R Studio version 1.2.5033.

## Results

### Feasibility

#### Recruitment

**Figure 2:**
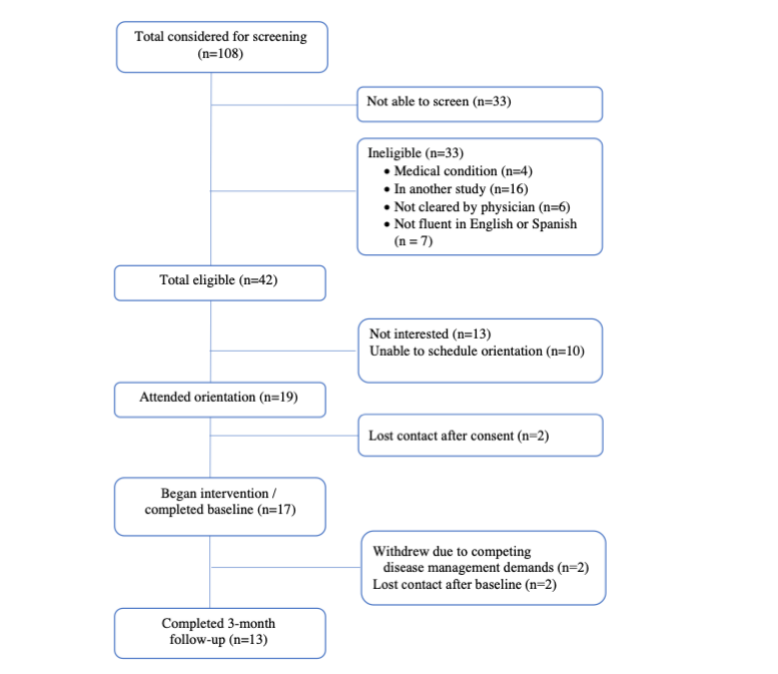
CONSORT (Consolidated Standards of Reporting Trials) diagram.

The flow of recruitment is shown in Figure 2. A total of 108 participants had visits scheduled in the clinic during the study period and were considered for participation based on chart review. Approximately one-third of the 108 patients were not screened in person because of missed appointments. Of the 75 patients screened, 42 met the eligibility criteria. The most common reasons for ineligibility were concurrent participation in another study (n=16), not speaking English or Spanish (n=7), and not receiving physician clearance for unsupervised exercise (n=6).

Of the 42 patients meeting eligibility criteria, 13 declined participation, and 10 expressed interest but were unable to schedule an orientation visit. The most common reason given for declining participation and being unable to schedule a visit was lack of time because of competing demands of diabetes management and prenatal care. Of the 19 participants who attended the orientation session and signed consent forms, 17 completed a baseline intervention visit and began the program. Only 2 participants withdrew following the orientation visit, citing the competing demands of diabetes management.

#### Baseline

Table 1 shows the baseline characteristics of the 17 participants who began the intervention. Participants were, on average, approximately 30 years old (mean 29.8, SD 3.85; range 23-36), and most were Hispanic (13/17, 76%). Nearly half (8/17, 46%) had no employment, and almost all (15/17, 88%) had Medi-Cal public insurance. Participants were a mix of primigravida and multiparous, with approximately two-thirds indicating that they had other children at home. Most (12/17, 71%) had T2D, and most were in the second trimester (mean 20 weeks and 5 days of gestation, SD 4.6). There was a broad range of metabolic markers, with HbA_1c_ ranging from 4.8 to 10%, and nonfasting glucose ranging from 96 to 179 mg/dL.

**Table 1:** Baseline characteristics of the study sample (N=17).

Demographics			Value
Age, mean (SD)			29.8 (3.85)
**Race and ethnicity, n (%)**			
	Non-Hispanic White		1 (6)
	Non-Hispanic Black		3 (18)
	Hispanic		13 (76)
**Language, n (%)**			
	English		13 (76)
	Spanish		4 (24)
**Employment, n (%)**			
	None		8 (46)
	Part-time		3 (18)
	Full-time		3 (18)
	No data		3 (18)
**Insurance type, n (%)**			
	Private		2 (12)
	Public		15 (88)
**Education, n (%)**			
	Less than high school		4 (24)
	High school		6 (36)
	Some college		6 (36)
	No data		1 (4)
**Marital status, n (%)**			
	Single		5 (29)
	Divorced/separated		1 (4)
	Married/partnered		10 (59)
	No data		1 (4)
**Other children at home, n (%)**			
	Any other children at home		10 (66)
	Children younger than 5 years at home		6 (40)
	Children aged 6-18 years at home		7 (47)
**Medical data**			
	**Diabetes type, n (%)**		
		Type 2	12 (71)
		Gestational	5 (29)
	BMI (kg/m^2^), mean (SD)		37.0 (4.1)
	Weight (lbs), mean (SD)		211.5 (39.2)
	Systolic blood pressure (mm Hg), mean (SD)		120.8 (17.2)
	Diastolic blood pressure (mm Hg), mean (SD)		70.7 (9.4)
	HbA_1C_^a^, mean (SD)		6.1 (1.4)
	Glucose (mg/dl) mean (SD)		112.5 (18.2)

^a^HbA_1c_: hemoglobin A_1c_.

#### Follow Up

Of the 17 participants who began the intervention, 13 (76%) completed a follow-up visit, and 12 (71%) continued to wear the Fitbit regularly after 8 weeks. A participant experienced technical difficulties and was not able to connect her Fitbit to the Fitabase system, so her data were not captured. Of the 4 participants lost to follow up, 2 withdrew during the intervention because of medical complications/early labor, whereas 2 chose to discontinue the study because of the competing demands of pregnancy. Participants’ time in the intervention ranged from 56 to 84 days, depending on the week of gestation upon enrollment and when they delivered.

Adherence was measured by examining how often participants wore their Fitbit monitors. There was great variability in the wear time among participants. Adherence ranged from 27% of days (23/84) to 100% of days (84/84), with a median wear day of 90% (mean 78%, SD 29%), indicating good adherence. However, full days of wear (≥600 min) were much more infrequent and ranged across participants from 0% to 86% of days, with a median of 50% (mean 48%, SD 32%). The median wear time on days with wear was 706 min (mean 749.1, SD 396.4). Limiting wear time to waking hours, median wear time was 643 min on days with any wear (mean 638.9, SD 282.9).

### Acceptability

Of the 13 participants who filled out consumer satisfaction questionnaires, 11 (85%) indicated that they found the counseling session quite/extremely helpful, and another 11 (85%) said that they felt moderately/very motivated to start exercising following the counseling session. The same number (11/13, 85%) indicated that they learned *a great deal* about physical activity in the counseling session, and 13 (13/13, 100%) said that they learned something new. With regard to the Fitbit, 11 (11/13, 85%) found it quite/extremely helpful in helping them increase their physical activity, and all but 1 participant (1/13, 8%) indicated that they wore it regularly. All but 1 participant (1/13, 8%) said that they were quite/extremely satisfied with the program overall, and 12 (12/13, 92%) said that they would probably/definitely recommend it to a friend.

A total of 10 participants completed a semistructured interview to provide additional feedback on the program. A common theme from the interviews was an appreciation for the low-burden/light-touch approach, with participants noting that the study encouraged them without feeling too pushy or intrusive:

pushed me harder… but didn’t put too much pressure on me… you guys were not invasive at all so I was able to go about my day.

Participant

Another noted that the study team “did not hover over me… I had my own space.”

Another participant said,

I also liked the feeling of having a support team without being pushed. I didn’t feel forced to be physically active.

Participant

Several participants mentioned that they appreciated not feeling *judged* or *ashamed* when they did not meet their goals.

Consistent with the results of the consumer satisfaction survey, another common theme was that the Fitbit was simple and easy to use. A participant shared:

I used the Fitbit every day. It was easy to track how many steps I had. Very user-friendly.

Participant

Several participants reported that they explored additional features of the Fitbit app that were not part of the intervention but had been mentioned by their physicians, such as tracking calories, hydration, and heart rate.

Participants also noted a number of features of the Fitbit app and wrist monitor that motivated them. Several participants enjoyed the push notifications from the Fitbit app, reporting daily step progress, or reminding them to move. A participant noted:

the updates I received letting me know how close or far I was from my goal also helped motivate me even more to try and achieve my goals.

Participant

Another appreciated the reinforcement the app provided when she met her goal:

it’s cool that the Fitbit shows a little rocket when you meet your goal… (my daughter) always wanted to see the rocket, so we walked around the park just to see the rocket.

Participant

Several participants also noted that it became more difficult to stay physically active as their pregnancies progressed. A participant noted,

I decreased my step goal toward the end of my pregnancy because it became harder to be physically active and meet my step goal.

Participant

Another participant recommended adding components to the intervention at the end to enhance motivation later in pregnancy. Another suggested a web-based support group or in a clinic.

### Change in Steps

**Figure 3:**
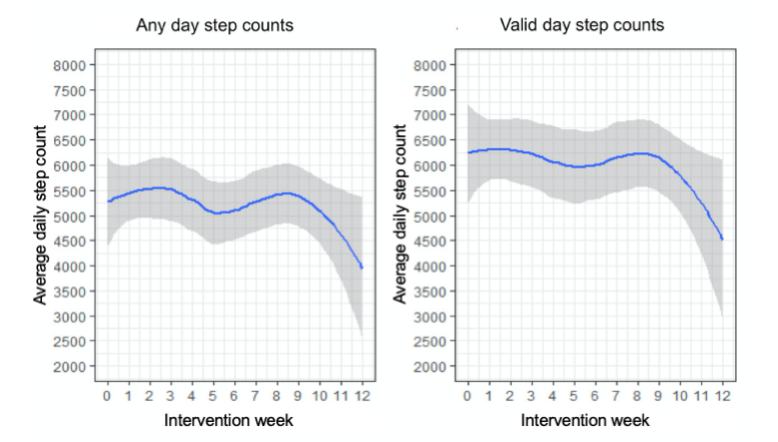
Average daily steps by week in the intervention.

At baseline, the mean number of steps per day was 5281 (SD 1846; Table 2). The average number of steps per day when only considering valid days was higher (mean 6122.1, SD 2356.2 steps per day). The average number of steps slightly increased from baseline to week 3 for both any wear days (mean 5504, SD 1846) and full valid days (mean 6268.6, SD 2081.3; Figure 3). For both any and valid days, the mean daily steps at week 6 were lower (mean 4657, SD 1751.9, and mean 5802.6, SD 2329.1, respectively) before rising again at week 9 (mean 5850.7, SD 2350.6, and mean 6770.4, SD 2563.3, respectively).

**Table 2:** Average daily steps by week in the study.

Period	Any wear		Valid full day	
	n	Mean (SD)	n	Mean (SD)
Baseline	16	5281 (1846)	15	6122 (2439)
Week 3	14	5504 (1984)	13	6269 (2166)
Week 6	13	4658 (1823)	11	5803 (2443)
Week 12	4	3888 (1551)	4	4191 (2228)

**Figure 4:**
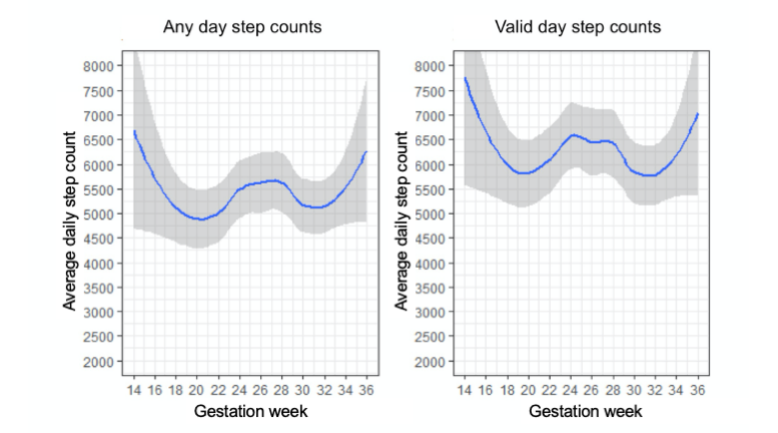
Average daily steps by gestation week.

On average, women entered the study at 21 weeks of pregnancy (mean 20.8, median 21, min/max 11/27). Figure 4 displays the average daily step counts by gestational week for any day step counts and full valid day step counts. All wear and valid full-day step counts were highest for women earliest in their pregnancies (14 weeks: mean 6208.1, SD 1324.1 for any wear; mean 7757.5, SD 2009.7 for valid days) and then dropped significantly by 19 weeks (any wear: mean 4973.4, SD 2543.8; valid days: 4334.7, SD 1991.6). Steps increased again toward the beginning of the third trimester (27 weeks, any wear: 6074.6 SD 1968.1; valid days: mean 6629.7, SD 2163.6) and then fell again as the third trimester progressed. Average step counts increased again between 33 and 36 weeks, but notably, only 2 women provided data beyond 33 weeks, contributing to more error in these estimates (Figure 4).

## Discussion

### Principal Findings

These results suggest good feasibility for a counseling- and wearable tracker-based physical activity intervention for pregnant women with diabetes. Of all eligible participants, most (29/42, 69%) expressed interest and enthusiasm for the study, and nearly half were enrolled (19/42, 45%). Retention and adherence were high, with a median wear time of 90% of days, and 76% (32/42) of participants completing a follow-up visit. The intervention also appeared highly acceptable to participants, with a large majority (39/42, 93%) saying that they were satisfied; 93% (39/42) saying that they would recommend the program, and 86% (36/42) indicating that they felt motivated to exercise more.

Changes in daily steps were modest and generally increased at the very beginning and then fell again, then rose again after the 6-week call, before falling once more at the end of the intervention; therefore by week 10, the mean daily steps were quite similar to what they were at baseline. Without a control group, it is difficult to know whether the intervention was successful in increasing the number of typical steps for this population. These results are similar to a recent study using Fitbits to increase physical activity in healthy pregnant women, which found small increases in steps in both the intervention and control groups (both received Fitbits) across 12 weeks [23].

Previous research has shown that activity tends to decrease during pregnancy, particularly from the second to the third trimester [13,14]. Schmidt et al [14] showed that although activity remained relatively consistent across the first and second trimesters, there were declines in both moderate activity and total energy expenditure between the second and third trimesters. A study by Pereira et al [15] showed that the decrease in physical activity during pregnancy was especially pronounced in insufficiently active women, as was also with the women in our study. Considering these trends, it is promising that women in this study kept relatively stable steps during the initial 8 to 10 weeks of the trial rather than showing a steady decrease. Data on changes in activity during pregnancy for women with diabetes, however, are scarce, and it is difficult to know how activity in this sample is compared with the larger population.

The greatest barrier to recruitment, apart from an ongoing trial in the same clinic with similar inclusion criteria, was enrollment under 28 weeks of gestation. As DAPP is a tertiary referral clinic, many women were referred late in gestation and did not have a regular primary care physician. Additionally, most women with GD did not receive a diagnosis until at least weeks 24-26 of gestation and thus were ineligible for the study by the time they were screened. Increasing physical activity at any stage of pregnancy is likely to confer benefits to both mother and child; however, earlier intervention may have the greatest clinical impact. This can only be made possible by larger, systems-level changes through greater access to prenatal care and/or universal promotion of physical activity programs to all pregnant women. In addition, despite the low burden of the intervention, of the 42 eligible patients, 23 did not enroll, mostly citing the competing demands of managing diabetes during pregnancy. This highlights the difficulty of recruiting this population and the need to reframe physical activity not as a competing demand but as a key component of diabetes management.

Conversely, retention was relatively high for a high-risk clinical population, particularly as some participated in their third trimester. A meta-analysis of physical activity interventions in pregnancy and risk of GD found that loss to follow up is a major barrier in these populations, reaching up to 33% attrition in some studies [9]. High retention in this intervention was likely owing to recruiting and delivering the intervention in a clinical setting where patients were expected to see their physician regularly and partnering with the attending physician to recruit and deliver the intervention. That said, following the initial goal-setting session, the entirety of the intervention was delivered remotely. Just 2 women stopped early because of medical complications/early labor, although these patients still provided consumer satisfaction data following delivery. Similarly, adherence was quite high, with participants wearing the Fitbit on approximately 90% of days they were in the intervention, although this decreased across the study. Russo et al [9] also found that adherence to physical activity interventions in pregnancy was highly variable and as low as 16%.

The high retention and adherence may have been owing to the high acceptability and light touch of the intervention. The most common theme in the follow-up interviews was an appreciation for the intervention not being invasive and that participants were allowed to go about their lives without feeling pushed or judged. This was facilitated by utilizing a face-to-face session to teach key behavior change strategies and then using the wearable tracker and app to reinforce these strategies throughout the intervention rather than relying on continued staff contact.

Although this approach was highly acceptable to participants, it is only ideal if it is also efficacious. Despite the fact that some more highly burdensome or *invasive* interventions may be less acceptable, it may also be the burden or the intrusion that leads to behavior change, and although only 1 participant specifically requested more staff contact, several noted a need for additional support during the third trimester. Future iterations of the intervention may need to incorporate greater intervention doses, particularly in the later stages of pregnancy, while still remaining noninvasive, particularly as this patient population is already burdened with tasks of diabetes management and prenatal care. This could be through greater staff contact, or still relying on technology to reinforce behavioral strategies but expanding to other channels (eg, texting) and with greater frequency. The need for this is reinforced by the fact that activity in this trial rose adjacent to contact with the study staff (baseline goal setting and 6-weeks check-in call).

### Strengths and Limitations

This study has several strengths, including the patient population recruited. Pregnant women with diabetes are a high-risk clinical population with a great need for lifestyle intervention. A common limitation in physical activity interventions in pregnancy is adherence and retention, but both of these factors remained relatively high through the progression of pregnancy. In addition, participants were racially and ethnically diverse, with Latinas comprising the majority. Several spoke only Spanish, and most (all but 2) relied on public insurance, representing a particularly high-risk, underserved population. In addition, all study visits were completed in exam rooms in the clinic, laying a foundation for an intervention with high potential for clinical implementation.

There are also important limitations to note. As noted previously, this was a single-arm trial, which limited our ability to pinpoint the effects of the intervention or to examine individual intervention components. Although diverse, the study population was difficult to recruit, and the sample size was small. Although parts of the intervention piggy-backed onto existing clinical protocols, such as including their daily steps in weekly glucose reports to their clinicians, clinicians reported a lack of resources for interpreting and responding to these reports, highlighting the need for multilevel interventions to successfully integrate interventions into clinical settings. In addition, because the use of accelerometers was discontinued, more detailed data on time in various activity intensities, including sedentary time, were not available. Despite these limitations, given the lack of research on this population, we feel that these findings lay a valuable foundation for the much-needed interventions for this high-risk group.

### Conclusions

Overall, these findings suggest that combined coaching- and mobile health–based physical activity intervention for pregnant women with diabetes is feasible and acceptable. A larger trial is needed to further explore the effect of wearable technology in physical activity interventions targeting pregnant women with diabetes. Future iterations of the intervention should include more staff contact and social support for the participants, especially during the later stages of pregnancy. Given the rapidly growing rates of diabetes in women of reproductive age, further development of such low-cost, low-burden interventions with potential for dissemination is vital to reducing complications and costs of diabetes in this high-risk population.

## Abbreviations

DAPP: Diabetes and Pregnancy Program
GD: gestational diabetes
HbA_1c_: hemoglobin A_1c_
T2D: type 2 diabetes
UCSD: University of California, San Diego
